# Identification of key genes controlling monoterpene biosynthesis of Citral-type *Cinnamomum bodinieri* Levl. Based on transcriptome and metabolite profiling

**DOI:** 10.1186/s12864-024-10419-7

**Published:** 2024-05-31

**Authors:** Qingyan Ling, Beihong Zhang, Yanbo Wang, Zufei Xiao, Jiexi Hou, Qingqing Liu, Jie Zhang, Changlong Xiao, Zhinong Jin, Yuanqiu Liu

**Affiliations:** 1 School of Soil and Water Conservation, Nanchang Institute of Technology, Jiangxi Provincial Engineering Research Center For Seed-Breeding and Utilization of Camphor Trees, Nanchang, China; 2 College of Forestry, Jiangxi Agricultural University, Jiangxi Key Laboratory of Subtropical Forest Resources Cultivation, Nanchang, China

**Keywords:** *Cinnamomum bodinieri* Levl., Citral, RNA-Seq, Metabolite, Geraniol

## Abstract

The citral-type is the most common chemotype in *Cinnamomum bodinieri* Levl (*C. bodinieri*), which has been widely used in the daily necessities, cosmetics, biomedicine, and aromatic areas due to their high citral content. Despite of this economic prospect, the possible gene-regulatory roles of citral biosynthesis in the same geographic environment remains unknown. In this study, the essential oils (EOs) of three citral type (B1, B2, B3) and one non-citral type (B0) varieties of *C. bodinieri* were identified by GC-MS after hydrodistillation extraction in July. 43 components more than 0.10% were identified in the EOs, mainly composed of monoterpenes (75.8–91.84%), and high content citral (80.63–86.33%) were identified in citral-type. Combined transcriptome and metabolite profiling analysis, plant-pathogen interaction(ko04626), MAPK signaling pathway-plant(ko04016), starch and sucrose metabolism(ko00500), plant hormone signal transduction(ko04075), terpenoid backbone biosynthesis (ko00900) and monoterpenoid biosynthesis (ko00902) pathways were enriched significantly. The gene expression of differential genes were linked to the monoterpene content, and the geraniol synthase (*CbGES*), alcohol dehydrogenase (*CbADH*), geraniol 8-hydroxylase-like (*CbCYP76B6-like*) and 8-hydroxygeraniol dehydrogenase (*Cb10HGO*) were upregulated in the citral-type, indicating that they were associated with high content of geraniol and citral. The activities of *CbGES* and *CbADH* in citral type were higher than in non-citral type, which was corroborated by enzyme-linked immunosorbent assay (*ELISA*). This study on the accumulation mechanism of citral provides a theoretical basis for the development of essential oil of *C. bodinieri*.

**Supplementary Information:**

The online version contains supplementary material available at 10.1186/s12864-024-10419-7.

## Introduction

The citral is very beloved worldwide for the sweet lemon fragrance, which is a food additive permitted and a raw material for the synthesis of violetone, widely used in the flavour, fragrance and perfume industry [1, 2]. Moreover, citral has anti-inflammatory, bactericidal, antioxidant, insecticidal activities and cardio protective potential properties [3–9]. Woody plants have large biomass generally, and it is significant if we can find tree species with high citral content. Natural citral was used to be extracted from the fruits of *Litsea cubeba* in China, but its fruit was particularly troublesome to pick and the fruit ripening season was also short [10]. The branches and leaves of *C. bodinieri* were found to be rich in citral, which will likely be a new source of natural citral. Unfortunately, *C. bodinieri* was divided into 13 types based on the principal components of the EOs, such as borneol-rich, camphor-rich, cadinol-rich, cymol-rich, nerolidol-rich, methyleugenol-rich, safrole-rich, citral-rich, and so on. Among these chemotypes, the camphor-rich and citral-rich are the two most common chemotype in *C. bodinieri* [11]. Why does same morphology *C. bodinieri* in the same geographical environment generate different essential oil compositions? A putative inherent genetic effect and differentiation may be existed; therefore, a comprehensive research of the mechanism of citral formation is highly desirable.

The research of *C. bodinieri* have focused on stress resistance [12], photosynthetic and fluorescence parameters [13], insect control [14], tissue culture and nursery technology [15]. Nevertheless, the formation mechanisms of the precious chemotype *C. bodinieri* EOs have rarely been studied. Citral is a monoterpenoid, and its synthesis pathways contain three steps. Firstly the terpenoid backbone were biosynthesized via mevalonate pathway and 2-C-Methyl-D-erythritol 4-phosphate pathway to generate isopentenyl-PP and dimethylallyl-PP. Secondly isopentenyl-PP and dimethylallyl-PP were catalyzed to geranyl-PP by geranyl diphosphate synthase [16]. Finally geranyl-PP were modified by different monoterpene synthases to produce various monoterpene compounds [17]. Geranial and neral were two isomers of citral, where does geranial and neral in *C. bodinieri* come from?

RNA-Seq technology was widely used in mining functional genes, probing active ingredient metabolic pathways and identifying key enzyme genes in *Cinnamomum camphora (C. camphora)* [18–21], *C. burmannii* [19]. Metabolites are the phenotypes in the central dogma of molecular biology, which can be regard as a fingerprint of the joint action of genes and the environment [22, 23]. In our study, essential oil of the leaves were extracted by hydrodistillation and indentified the main components by GC-MS. Transcriptomics and volatile metabonomics in the citral-type and non-citral type varieties of *C. bodinieri* were performed. In addition, the genes related to the biosynthesis of citral were corroborated by qRT-PCR and ELISA. The elucidation of the formation mechanism of citral would lay the theoretical foundation for improving the yield and quality of essential oil of citral-type *C. bodinieri.*

## Materials and methods

### Plant material and reagent

Three citral-type (B1, B2, B3) and one non-citral type (B0) varieties of 15-year-old *C. bodinieri*, were sourced from forestry in the Tianzhu County, Qiandong nan Miao and Dong Autonomous Prefecture, Guizhou province, China (Latitude: 26° 97′ N, Longitude: 109° 10′ E) in July 2022. The plants were authenticated by professor Zhinong Jin, and deposited in the gene bank of Nanchang institute of technology, and the voucher numbers were *C. bodinieri*-GZ/TZ/028 (B1); *C. bodinieri*-GZ/TZ/029 (B2); *C. bodinieri*-GZ/TZ/031 (B3); *C. bodinieri*-GZ/TZ/041 (B0). The citral-type *C. bodinieri* used in our study were validated as Jiangxi provincial improved tree varieties after specificity, consistency and stability tests (http://ly.jiangxi.gov.cn/art/2023/10/16/art_68735_4629779.html).

### Essential oils isolation and analysis

Essential oils were extracted from the leaves of 15-year old cuttings of the same height of three citral-type (B1, B2, B3) and one non-citral type (B0) varieties in July 2022, three sample plants were randomly selected as replicates for each variety. 400 g leaves and 2 L distilled water were placed in a hydro-distilled modified Clevenger apparatus [24], then distillated for 1 h. Calculate the essential oils yield based on the following formula:

$${\rm{Y}}\,{\rm{ = }}\,{\rm{(}}{{\rm{M}}_{\rm{1}}}\,{\rm{/}}\,{{\rm{M}}_{\rm{2}}}{\rm{)}}\,{\rm{ \times }}\,{\rm{100}}\,{\rm{\% }}$$

where: Y is the leaf oil yield (%); M_1_ is the weight of essential oils; M_2_ is the weight of leaves.

Analyses of EOs compounds were performed by GC-MS (Agilent 7890B-5975 C, USA), referring to our previous study [7]. The HP-5MS (30 m×250 μm×0.25 μm) column was used. The electron impact of the mass spectrometer was 70 eV, the scanning mass range was set at 50 ~ 650 m/z, the scanning rate was set at 0.5 times/s, the temperature of the conductor was 250℃, the temperature of the ion source was 230℃, the temperature of the quadrupole was 150℃, and the doubling voltage was 1,200 V. Helium was used as the carrier gas (the flow rate was 1.2 mL/min), and 0.1 µL was injected (the separation ratio was 20 : 1). The oven temperature program conditions were as follows: an initial temperature of 80℃ for 5 min with a solvent delay of 3 min, followed by a gradual increase to 120℃ at a rate of 2.5 ℃/min, where it was held for 1 min, and then to 240℃ at a rate of 20 ℃/min for 5 min. The total run time was 60 min. Essential oils were diluted with methanol (1%), filtered, and auto-sampled.

The literature retention index (RI) was determined from the NIST library (https://webbook.nist.gov/chemistry/). The experimental retention index (RI) was determined by analyzing the samples with n-alkane C7-C40 standards under the same chromatographic conditions by injecting the samples, calculating and comparing RI, identifying as the same compound when RI in the experiment and in the literature were within 30:

$${\rm{RI}}\,{\rm{ = }}\,{\rm{100}}\left[ {{{{\rm{lo}}{{\rm{g}}_{{\rm{10}}}}{X_{\rm{i}}}\, - \,{\rm{lo}}{{\rm{g}}_{{\rm{10}}}}{X_{\rm{n}}}} \over {{\rm{lo}}{{\rm{g}}_{{\rm{10}}}}{X_{{\rm{n + 1}}}}\, - \,{\rm{lo}}{{\rm{g}}_{{\rm{10}}}}{X_{\rm{n}}}}}\,{\rm{ + }}\,n} \right]$$

where: X is the retention time of the unknown compound; Xn and Xn + 1 are the retention times of the corresponding before and after n-alkanes (Xn < Xi < Xn + 1).

### RNA sequencing and data analysis

Twelve RNA samples (B0, B1, B2, B3 groups with three replicates) were extracted by the RNeasy Plant Mini Kit (Qiagen, Germany). Sequencing libraries were generated using NEBNext® Ultra™ RNA Library Prep Kit and library quality was inspected by Agilent Bioanalyzer 2100, then sequenced using the Illumina platform, finally, sequence data was filtered to get clean data. Over 83% of filtered reads per sample were mapped to the *C. camphora* reference genome (GWHBGBX00000000) [25] (Table S1). Final unigenes were aligned to the NCBI non-redundant (Nr), KEGG pathway database, Gene ontology (GO), KOG database, and Swiss-Prot protein database. Feature Counts v1.6.2 was used to calculate Fragments Per Kilobase of Transcript Per Million Mapped Reads (FPKM). The thresholds for significantly differential expression were set at *p* ≤ 0.01 and |log2(FoldChange)| ≥ 1. The raw sequence data has been submitted to the NCBI (PRJNA970043). PCA (principal component analysis), Heatmap (Visualization map), Inter-sample correlation plot and OPLS-DA were performed by statistics function within R (www.r-project.org).

### Candidate genes confirmation

To confirm candidate genes related to formation of citral, the DEGs related to terpenoid backbone biosynthesis and monoterpenoid biosynthesis pathways were selected for quantitative real-time PCR (qRT‑PCR) [26], and three replications were done for each validation. Amplification consisted in an initial denaturation step at 95℃ for 5 min, followed by 40 cycles of denaturating at 95℃ for 10 s, 60℃ annealing temperature for 30 s. The specificity of primer was verified through a melting curve analysis at the end of the qRT‑PCR (0.5℃ ramping for 10 s, from 60℃ to 95℃). Normalization of qRT-PCR expression analysis of target genes was achieved through internal reference gene *ACTIN* and the primers were designed using the NCBI Primer-BLAST designing tool (Table S2). Relative gene expression was calculated using the 2^−△△ct^ method and the graphic was statistically analyzed by Origin(version 2023b, USA).

### Enzyme viability assay

The geraniol synthase (*CbGES*) and alcohol dehydrogenase (*CbADH*) enzyme activities of twelve samples (B0, B1, B2, B3 groups with three replicates) were determined by an enzyme-linked immunosorbent assay (ELISA) kit specific for plant(Mlbio, Shanghai, China) according to the manufacturer’s instructions. Double antibody sandwich method was used in the kit assay plant *CbGES* and *CbADH* level in the sample. At first, the capture antibody was encapsulated on a solid phase carrier, and antigen was added to bind, and the re-added detection antibody binded to the antigen, turned into antibody-antigen-enzyme-antibody complex, added TMB substrate and became blue color at HRP enzyme-catalyzed, then the color transformed to yellow by the action of acid. Finally, the absorbance (OD) was measured at 450 nm with an enzyme standard meter, and the enzyme activity by comparing the OD of the samples to the standard curve was calculated.

## Result

### Essential oils yield of citral-type and non-citral type *C. Bodinieri* leaves

**Fig. 1:**
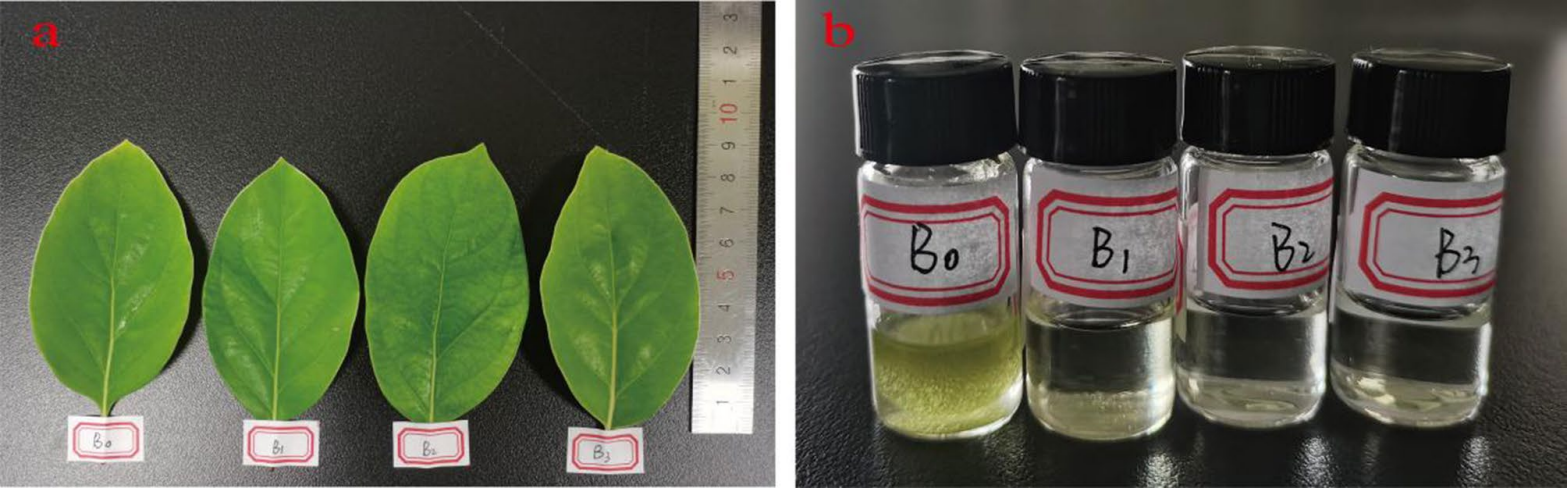
Citral-type and non-citral type samples of *C. bodinieri.* (**a**) Two chemotypes of *C. bodinieri* leaves. (**b**) Two chemotypes of *C. bodinieri* leaves EOs

The citral-type and non-citral type leaves were the same in morphology. The citral-type leaves EOs were transparent, whereas the non-citral type leaves EOs appeared camphor crystals precipitated and yellowish (Fig. 1). The oil yield ranged from 0.35 to 0.89% (w/w FW) and from 0.82 to 1.92% (w/w DW), respectively. The EOs yields of citral-type leaves were higher than the non-citral type (*p* ≤ 0.01) (Table 1).

**Table 1:** Essential oils content of the *C. bodinieri* leaves (%)

Different varieties	B1	B2	B3	B0
Fresh weight EOs content (%)	0.80 ± 0.05^a^	0.89 ± 0.09^a^	0.87 ± 0.17^a^	0.35 ± 0.18^b^
Dry weight EOs content (%)	1.77 ± 0.06 ^a^	1.92 ± 0.08^a^	1.78 ± 0.15^a^	0.82 ± 0.16^b^
EOs characteristics	Transparent	Transparent	Transparent	Precipitation of crystals and yellowish

*Note* Data followed by the different lowercase letters indicated significant differences according to Duncan’s test with 1% significance (*p* ≤ 0.01)

### Essential oils composition of citral-type and non-citral type *C. Bodinieri* leaves

The GC-MS experiment identified B2 variety attained 94.17% (14 constituents) and B1 variety 93.87% (14 constituents), followed by B0 variety 92.30% (34 constituents), B3 variety 90.56% (15 constituents). Two geometric isomers of citral, geranial and neral were dominated in the EOs composition in leaves of the citral-type *C. bodinieri* (B1, B2 and B3). In addition, unsaturated citral Z-isocitral and E-isocitral were existed frequently. The four dominant components ranged from 81.65 to 88.23% (Table 2). In the non-citral type *C. bodinieri* (B0), camphor (46.12%) was the most abundant, and borneol (14.67%) followed. Oxygenated monoterpenes dominated in the chemical composition of B0-B3 *C. bodinieri* EOs with proportions of 66.90%, 91.64%, 91.80% and 87.31%, respectively.

**Table 2:** Essential oils composition in leaves of the *C. bodinieri*

No	RI	RI	Compounds	Molecular	Percent Composition			
	(lit)	(exp)		Formula	B0	B1	B2	B3
1	921	923	Thujene	C_10_H_16_	0.20	-	-	-
2	937	938	α-Pinene	C_10_H_16_	2.43	-	0.24	0.19
3	951	953	Camphene	C_10_H_16_	1.53	-	-	-
4	972	975	Sabinene	C_10_H_16_	0.43	-	-	-
5	981	978	β-Pinene	C_10_H_16_	1.38	-	-	-
6	1026	1025	Limonene	C_10_H_16_	2.93	0.20	0.24	0.20
7	1030	1029	Eucalyptol	C_10_H_18_O	0.97	0.41	0.55	0.41
8	1075	1081	trans-Sabinene Hydrate	C_10_H_18_O	0.20	-	-	-
9	1097	1095	α-Terpinolene	C_10_H_18_O	0.40	-	-	-
10	1101	1098	Linalool	C_10_H_18_O	2.19	0.22	0.24	0.29
11	1144	1141	Camphor	C_10_H_16_O	46.12	-	-	-
12	1147	1153	6-Octenal, 7-methyl-3-methylene-	C_10_H_16_O	-	0.24	0.29	0.23
13	1158	1156	Citronellal	C_10_H_18_O	-	0.23	0.28	0.27
14	1165	1165	Z-isocitral	C_10_H_16_O	-	0.34	0.64	0.28
15	1167	1169	Borneol	C_10_H_18_O	14.67	-	-	-
16	1172	1171	4-Terpinenol	C_10_H_18_O	0.70	-	-	-
17	1184	1179	E-isocitral	C_10_H_16_O	-	0.68	1.26	0.74
18	1206	1181	α-Terpineol	C_10_H_18_O	1.20	-	-	-
19	1217	1222	Citronellol	C_10_H_20_O	-	0.59	0.82	0.87
20	1245	1247	Neral	C_10_H_16_O	-	37.13	37.18	32.57
21	1250	1246	Geraniol	C_10_H_18_O	-	1.10	1.39	0.99
22	1276	1277	Geranial	C_10_H_16_O	-	48.19	49.15	48.06
23	1286	1285	Safrole	C_10_H_10_O_2_	0.40	-	-	-
24	1355	1346	Geranic acid	C_10_H_16_O_2_	-	2.51	-	2.60
25	1399	1403	Caryophyllene	C_15_H_24_	1.52	0.60	0.56	0.79
26	1454	1454	Humulene	C_15_H_24_	1.75	-	-	-
27	1472	1475	Germacrene D	C_15_H_24_	0.64	-	-	-
28	1486	1489	Bicyclogermacrene	C_15_H_24_	3.80	-	-	-
29	1519	1518	delta-Cadinene	C_15_H_24_	0.20	-	-	-
30	1537	1540	Elemol	C_15_H_26_O	0.30	-	-	-
31	1550	1548	Germacrene B	C_15_H_24_	0.20	-	-	-
32	1562	1566	E-Nerolidol	C_15_H_26_O	0.74	-	-	-
33	1576	1573	Spathulenol	C_15_H_24_O	2.00	-	-	-
34	1578	1578	Caryophyllene oxide	C_15_H_24_O	1.20	1.43	1.33	2.07
35	1587	1560	Viridiflorol	C_15_H_26_O	0.20	-	-	-
36	1591	1595	Guaiol	C_15_H_26_O	0.30	-	-	-
37	1606	1607	Humulene epoxide II	C_15_H_24_O	0.40	-	-	-
38	1644	1641	Isospathulenol	C_15_H_24_O	0.80	-	-	-
39	1662	1660	Neointermedeol	C_15_H_26_O	1.10	-	-	-
40	1666	1668	Bulnesol	C_15_H_26_O	0.20	-	-	-
41	1689	1692	Schyobunol	C_15_H_26_O	0.10	-	-	-
42	1705	1705	(Z, Z)-2,6-Farnesol	C_15_H_26_O	0.80	-	-	-
43	1720	1721	(E, Z)-2,6-Farnesal	C_15_H_24_O	0.30	-	-	-
Amount of chemical compounds					34	14	14	15
Total identified constituents					92.30	93.87	94.17	90.56
Hydrocarbon monoterpenes (HM) 1,2,3,4, 5,6.					8.90	0.20	0.48	0.39
Oxygenated monoterpenes (OM)7,8,9,10,11,12,13,14,15, 16,17,18,19,20,21,22,23,24.					66.90	91.64	91.80	87.31
Hydrocarbon sesquiterpenes (HS)25,26,27,28,29,31.					8.10	0.60	0.56	0.79
Oxygenated sesquiterpenes (OS) 30,32,33,34,35,36,37,38,39,40,41,42,43.					8.40	1.43	1.33	2.07

*Note* More than 0.10% were identified in the EOs. —not detected

### Transcriptome analysis of citral-type and non-citral type *C. Bodinieri* leaves

To identify the genes involved in citral-type *C. bodinieri* monoterpene biosynthesis, RNA-Seq was performed on three citral-type (B1, B2, B3) and one non-citral type (B0) varieties. A total of 12 cDNA libraries were constructed, comprising 558.83 million raw reads. After quality control, 538.02 million high-quality clean reads, including 80.7 Gb of nucleotide sequences.Furthermore, the percentage of Q30 base in each sample was not less than 92.27%. Over 83.26% of filtered reads per sample were mapped to the *C. camphora* reference genome (Table S1). 5,560 unigenes were annotated in NR, 4,697 in Pfam, 4,357 in Swiss-Prot, 418 in TF, 4,285 in KEGG, 4,842 in GO, 5,555 in TrEMBL and 2,868 in KOG databases (Table S3).The DEGs based on Kyoto Encylopedia of Genes and Genomes (KEGG) and Gene Ontology (GO) were analyzed (Figures S1 and S2).

**Fig. 2:**
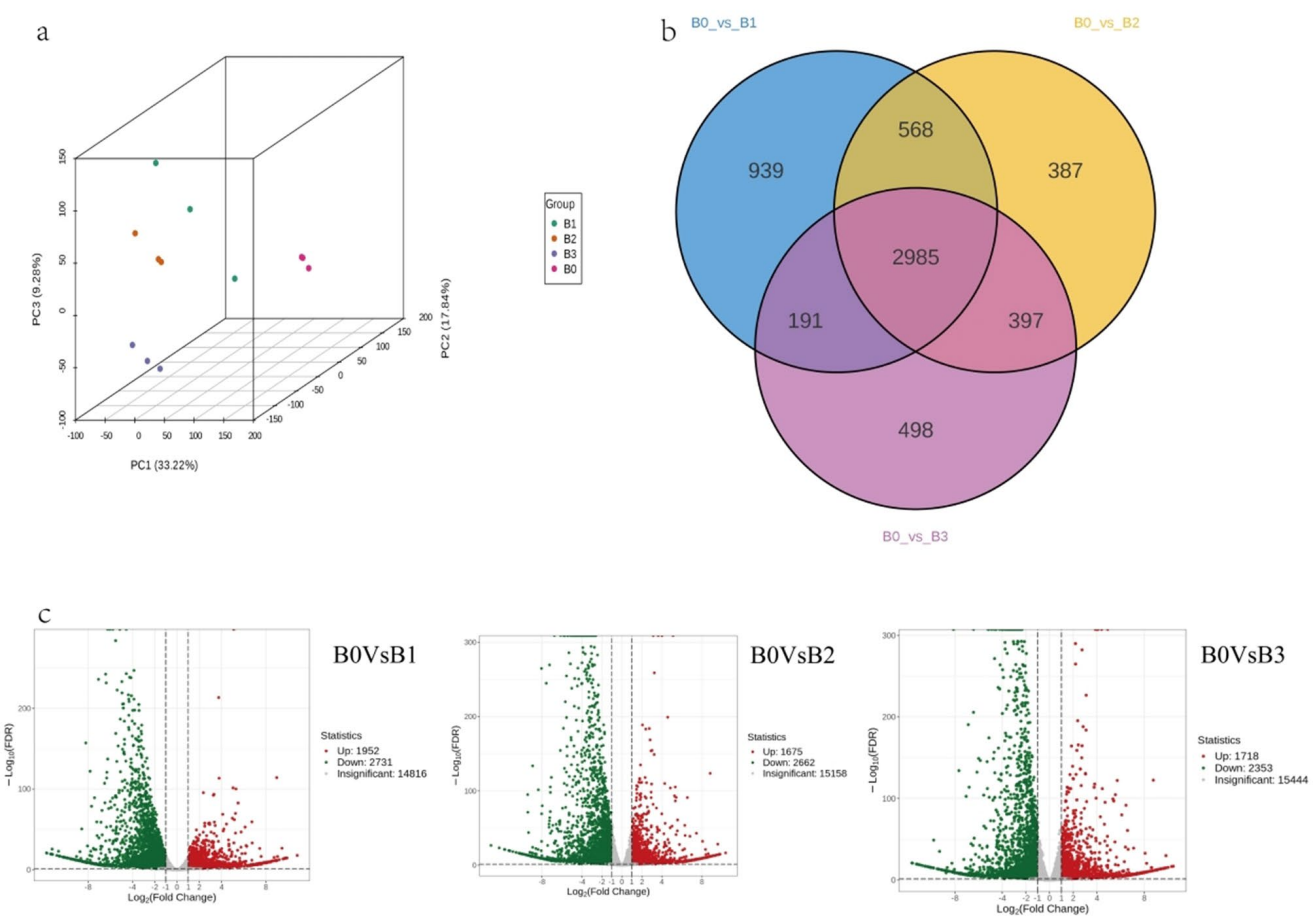
Transcriptomic analysis. (**a**) Principal component analysis (PCA) score plots. (**b**) Venn diagram of DEGs. (**c**) Volcano plots of differentially expressed genes (DEGs)

Three citral-type (B1, B2, B3) and one non-citral type (B0) varieties were clearly separated in principal component analysis (PCA) score plots (Fig. 2a). In B0 Vs B1, B0 Vs B2 and B0 Vs B3 volcano plots, 8337 DEGs (1952 upregulated and 2731 downregulated), 3710 DEGs (1675 upregulated and 2662 downregulated), and 6202 DEGs (1718 upregulated and 2353 downregulated) were displayed respectively (Fig. 2c). Venn diagram showed that 2985 DEGs were identified between citral-type and non-citral type, which may be associated with lemon and camphor fragrance differences (Fig. 2b).

### Screening of genes related to synthesis of monoterpenoids

**Fig. 3:**
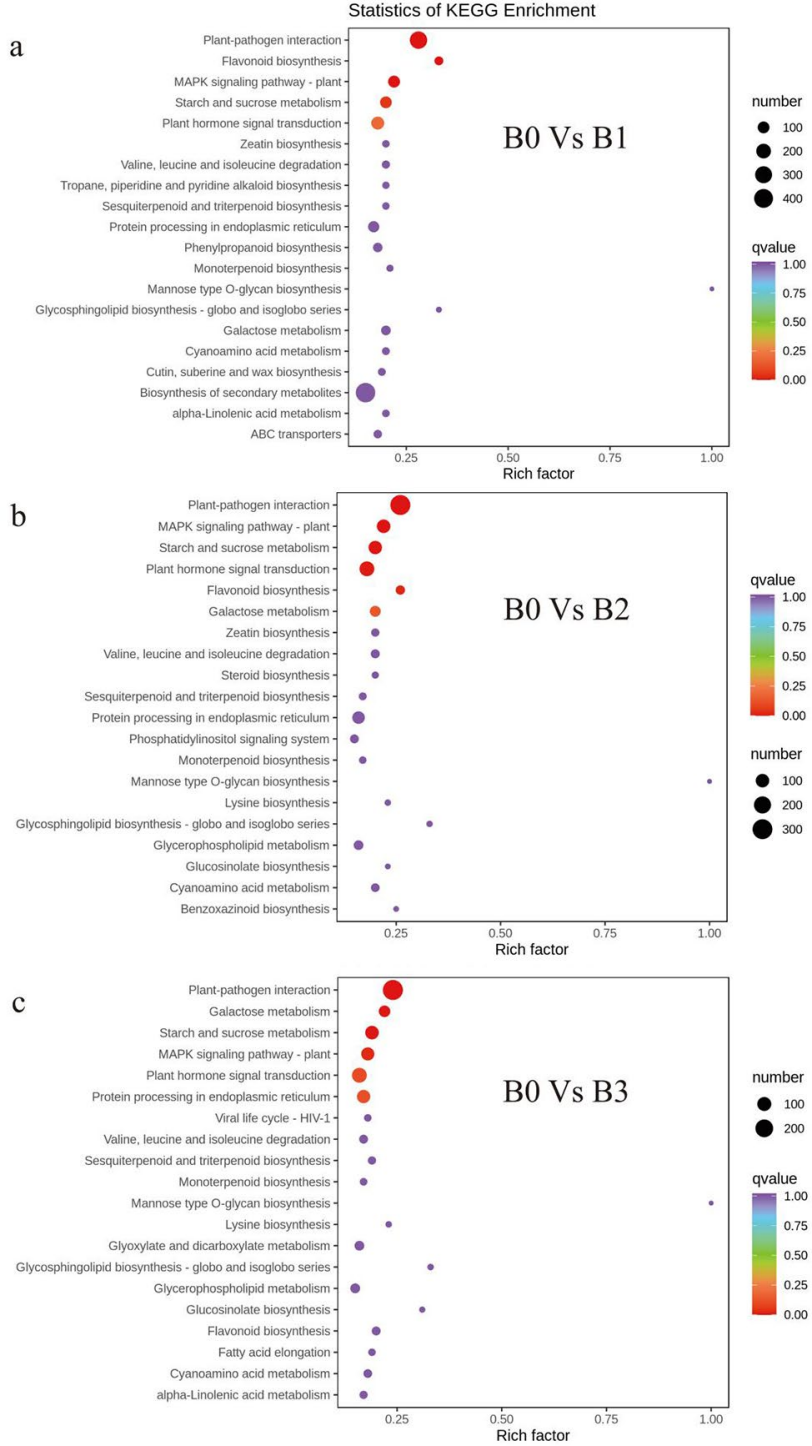
KEGG enrichent scatter plot of DEGs. (**a**) B0 vs. B1. (**b**) B0 vs. B2. (**c**) B0 vsB3

KEGG enrichment scatter plot of DEGs showed that plant-pathogen interaction (ko04626), MAPK signaling pathway-plant (ko04016), starch and sucrose metabolism (ko00500) and plant hormone signal transduction (ko04075) were enriched significantly (Fig. 3).

**Fig. 4:**
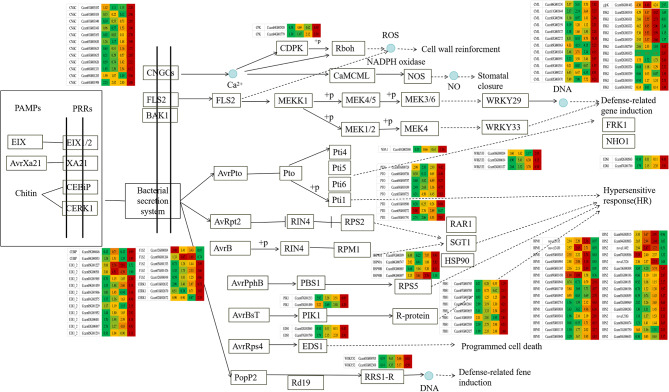
Differential expression of genes involved in plant-pathogen interaction pathway in citral-type and noncitral-type of *C. bodinieri*, designated as B1, B2, B3 and B0, respectively. Expression values were presented as (FPPM + 1) normalized log_2_-transformed counts

**Fig. 5:**
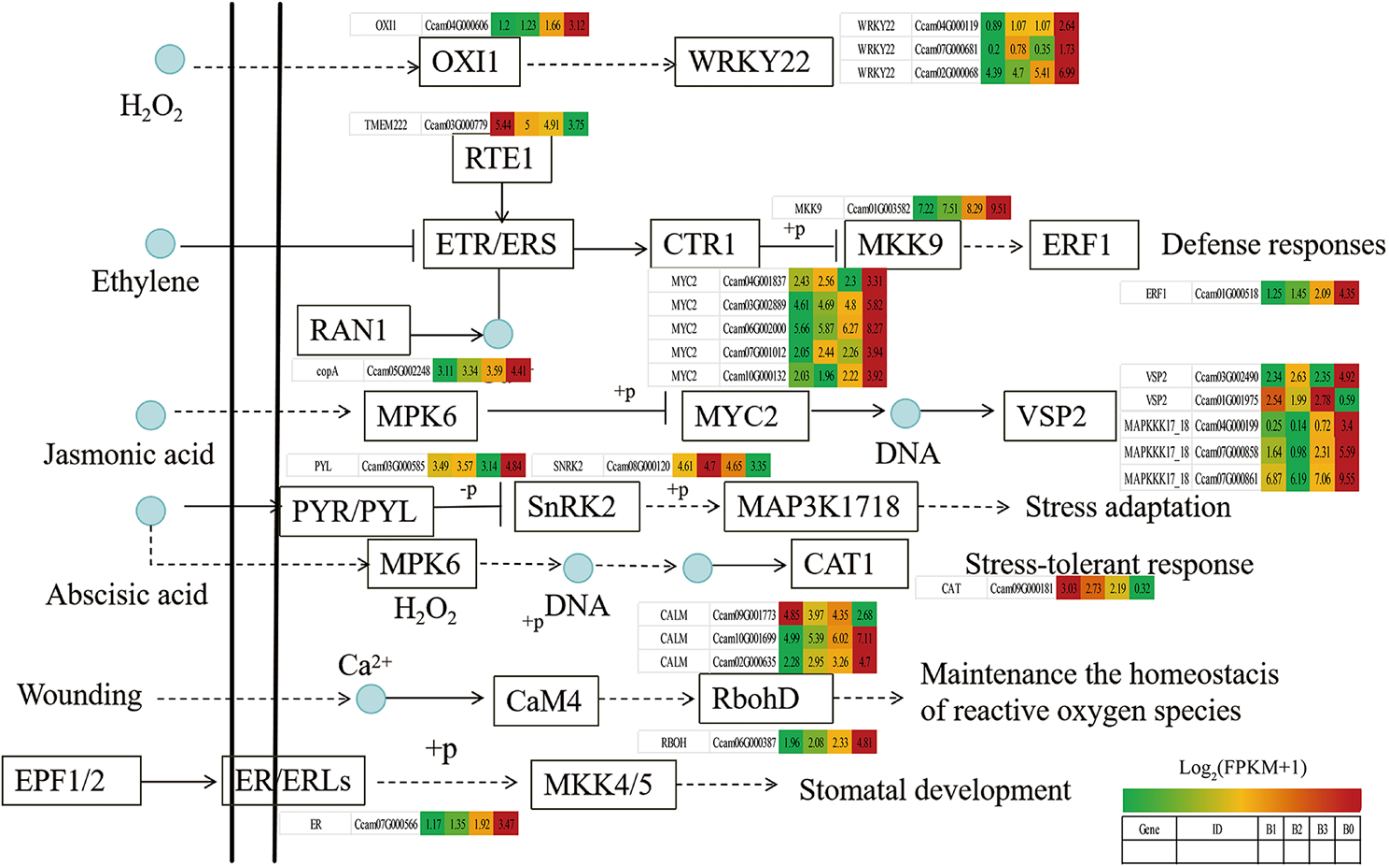
Differential expression of genes involved in MAPK signaling pathway-plant pathway in citral-type and noncitral-type of *C. bodinieri*

**Fig. 6:**
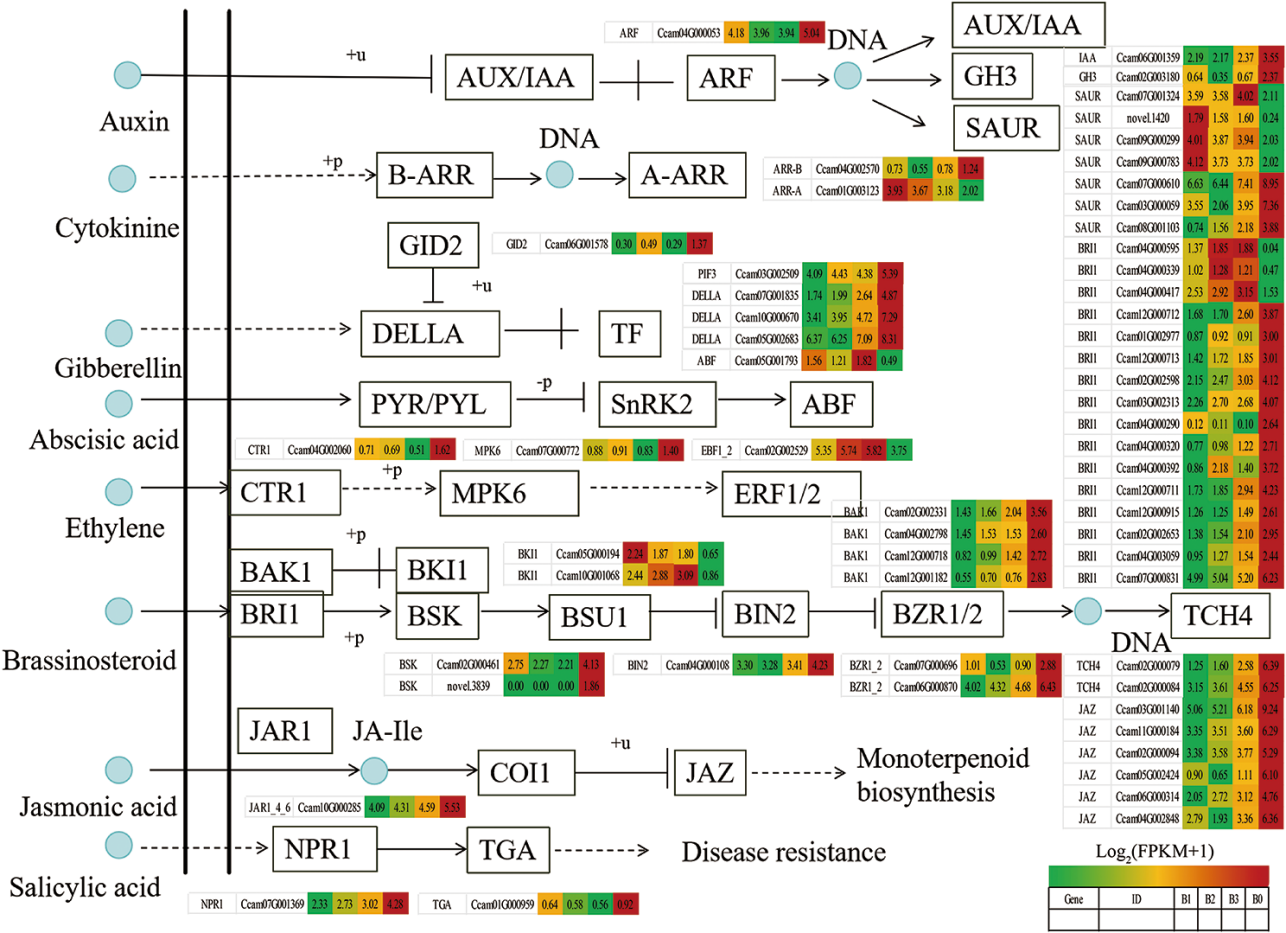
Differential expression of genes involved in plant hormone signal transduction pathway in citral-type and noncitral-type of *C. bodinieri*

The transcriptional expression of ko04626, ko04016 and ko04075 pathway genes were investigated by preparing heat maps (Figs. 4, 5 and 6).As expected, only several putative nucleoside-diphosphate kinase (ndk), catalase (CAT), serine/threonine-protein kinase SRK2 (SNRK2), EIN3-binding F-box protein (EBF1_2), transmembrane protein 222 (TMEM222), glycerol kinase(glpk), BRI1 kinase inhibitor 1 (BKI1), two-component response regulator ARR-A family (ARR-A), ABA responsive element binding factor (ABF) and phenylpyruvate tautomerase (MIF) genes were up-regulated in B1, B2 and B3 relative to B0, respectively. Most other genes were signifcantly down-regulated in B1, B2 and B3 relative to B0, respectively (Figs. 4–6, Table S4-S6). It is indicated that most genes in plant-pathogen interaction (ko04626), MAPK signaling pathway-plant (ko04016) and plant hormone signal transduction (ko04075) pathways were actively expressed in camphor-type *C. bodinieri*, which might be related to the stronger anthelmintic and antibacterial properties of the camphor.

**Fig. 7:**
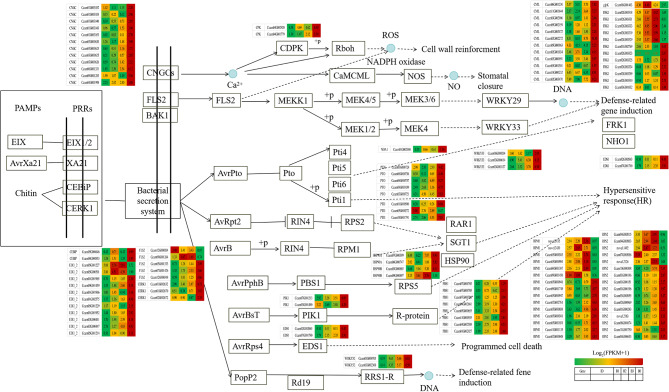
Differential expression of genes involved in starch and sucrose metabolismpathway in citral-type and noncitral-type of *C. bodinieri*

The transcriptional expression of ko00500 pathway genes was also investigated by preparing heat maps (Fig. 7). The starch synthase (glgA), UTP–glucose-1-phosphate uridylyltransferase (galU) and ectonucleotide pyrophosphatase/phosphodiesterase family member 1/3 (ENPP1_3) were signifcantly up-regulated in B1, B2 and B3 relative to B0, respectively. Five trehalose 6-phosphate synthase/phosphatase (TPS), glucan endo-1,3-beta-glucosidase 1/2/3 (GN1_2_3), two glucose-1-phosphate adenylyltransferase (glgC) and seven alpha-amylase (amyA) were signifcantly down-regulated in B1, B2 and B3 relative to B0, respectively (Fig. 7, Table S7). We hypothesized that the active expression of these genes involved in starch and sugar metabolism were related to the need for carbohydrates for secondary metabolism in *C. bodinieri*.

However, monoterpene was the largest proportion of *C. bodinieri* essential oils composition (Table 2), so we focused on genes related to monoterpene synthesis when screening for related genes. Four monoterpenoid synthesis pathways were further screened, terpenoid backbone biosynthesis (ko00900), monoterpenoid biosynthesis (ko00902), pinene, camphor and geraniol degradation (ko00907) and sesquiterpenoid and triterpenoid biosynthesis (ko00909).

**Fig. 8:**
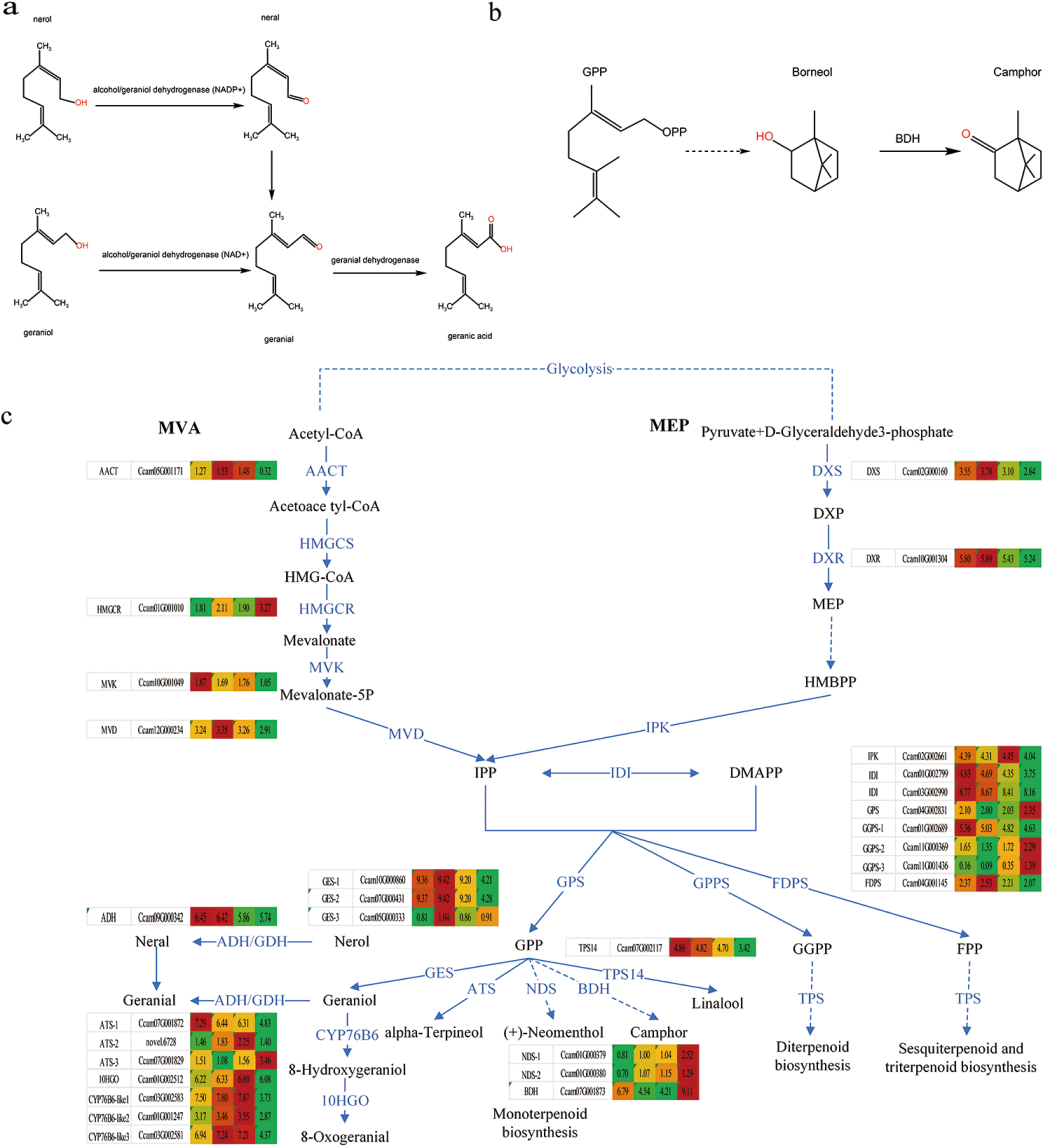
Exploration of the monoterpene biosynthesis pathway in *C. bodinieri.* (**a**) Citral synthesis pathway diagram. (**b**) Camphor synthesis pathway diagram. (**c**) Heat map of expression of key genes for the formation of citral and camphor

Geraniol and nerol were catalyzed to geranial and neral by geraniol dehydrogenase (NAD^+^) or alcohol dehydrogenase(NADP^+^), respectively. Next, geranial was catalyzed to geranic acid by geranial dehydrogenase (Fig. 8a). Additionally, geranyl diphosphate (*GPP*) was cycled and hydrolyzed to borneol, and then borneol was oxidized to camphor by borneol dehydrogenase *(BDH*) (Fig. 8b).

All monoterpene production was dependent on terpenoid backbone biosynthesis, therefore, genes encoding acetyl-CoA C-acetyltransferase (*CbAACT*), 1-deoxy-D-xylulose-5-phosphate synthase (*CbDXS*), 1-deoxy-D-xylulose-5-phosphate reductoisomerase (*CbDXR*), iphosphomevalonate decarboxylase (*CbMVD*), isopentenyl phosphate kinase (*CbIPK*), isopentenyl-diphosphate Delta-isomerase (*CbIDI*), geranyl diphosphate synthase (*CbGPS*), geranylgeranyl diphosphate synthase, type II (*CbGPPS*) and farnesyl iphosphate synthase (*CbFDPS*) were identified. These key genes in MEP and MVA pathways were expressed actively, with high expression in both citral-type and non-citral type of *C. bodinieri*, favouring the synthesis of monoterpene synthetic precursors (Fig. 8c). In the monoterpenoid biosynthesis pathway, alpha-terpineol synthase (*CbATS*), (3 S)-linalool synthase (*CbTPS14*), neomenthol dehydrogenase (*CbNDS*), geraniol 8-hydroxylase-like (*CbCYP76B6-like*), 8-hydroxygeraniol dehydrogenase(*Cb10HGO*) and borneol diphosphate synthase (*CbBDH*) were identified. Genes encoding of the geraniol metabolic pathway was identified including geraniol synthase (*CbGES*) and alcohol dehydrogenase (*CbADH*) (Fig. 8c).

Among these genes, the expression of *CbAACT*, *CbDXS*, *CbDXR*, *CbMVD*, *CbIPK*, *CbIDI*, *CbIPPI*, *CbGPPS*, *CbFDPS*, *CbGES*, *CbADH*, *CbCYP76B6-like*, *Cb10HGO* and *CbATS* were significantly higher in the citral-type. The neomenthol dehydrogenase (*CbNDS*) and borneol diphosphate synthase (*CbBDH*) showed significantly higher expression in non-citral type in comparison to citral-type leaf expression.

### Confirmation of candidate genes related to formation of citral via qRT‑PCR

**Fig. 9:**
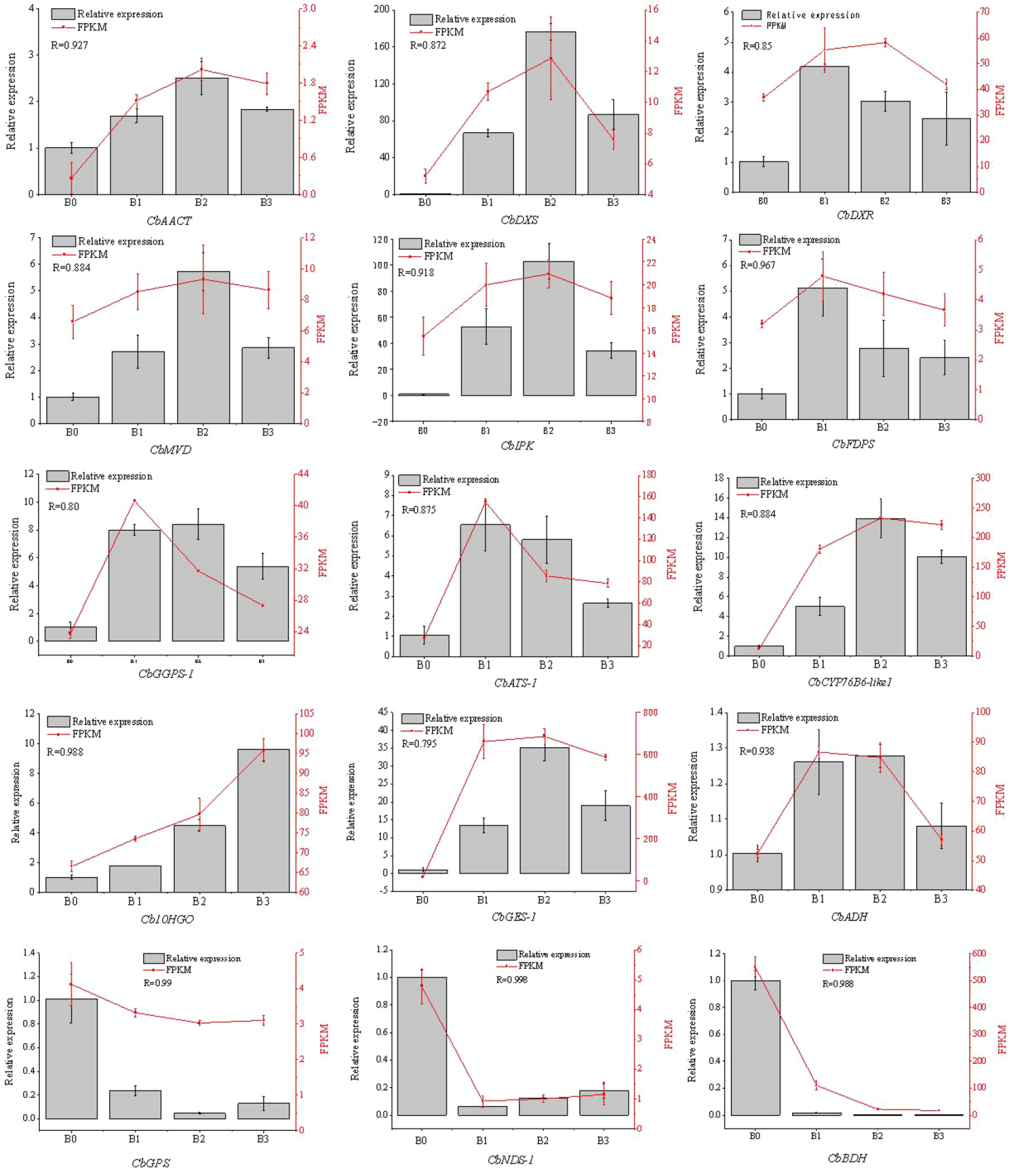
The expression of 15 genes related to citral and camphor formation was determined by qRT-PCR. *Note*: R mean pearson correlation coefficients between the relative expression and FPKM

We further selected 8 genes in terpenoid backbone biosynthesis pathway (*CbAACT*, *CbDXS*, *CbDXR*, *CbMVD*, *CbIPK*, *CbFDPS*, *CbGPPS* and *CbGPS*), 7 genes in monoterpenoid biosynthesis pathway (*CbATS*, *CbCYP76B6-like*, *Cb10HGO*, *CbGES*, *CbADH*, *CbNDS* and *CbBDH*), to validate the transcriptomic results. The expression of these unigenes observed by qRT-PCR were strongly supported the RNA-Seq value (Fig. 9). The expression of *CbAACT*, *CbDXS*, *CbDXR*, *CbMVD*, *CbIPK*, *CbFDPS*, and *CbGPPS* in citral-type was significantly higher than that in non-citral type. This result was corroborated with the high content of monoterpenes (hydrocarbon and oxygenated) in the citral-type (Table 2). The high expression of these genes provided abundant precursors for the synthesis of monoterpenes. In addition, the expression of *CbGPS* was lower in citral-type, whereas the *CbGPS* was a key gene of monoterpenes synthesis universal precursor geranyldiphosphate (GPP). Moreover, the expression of *CbATS*, *CbCYP76B6-like*, *Cb10HGO*, *CbGES*, *CbADH* in citral-type were significantly higher than that in non-citral type. Notably, the relative expression of *CbNDS* and *CbBDH* in non-citral type were similar to their FPKM (*R* = 0.998 and 0.988) (Fig. 9).

### Enzyme activity of *CbGES* and *CbADH* in citral-type and non-citral type

**Fig. 10:**
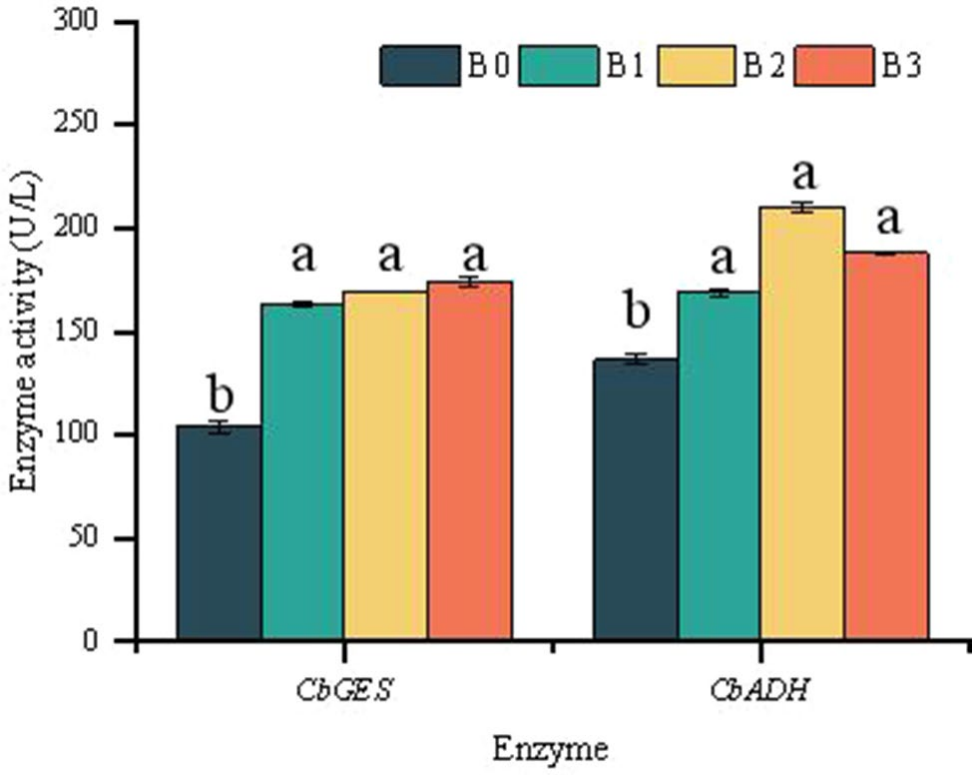
Enzyme activity of the geraniol synthase (*CbGES*) and alcohol dehydrogenase (*CbADH*) in the citral-type (B1, B2, B3) and non-citral type (B0) by ELISA (*p* < 0.01)

Transcriptome analyses of *C. bodinieri* and qRT-PCR assays showed that citral content was related to the gene expression of *CbGES* and *CbADH*. An enzyme-linked immunosorbent assay (ELISA) can be used for quantitative measurement of protein. The geraniol synthase (*CbGES*) enzyme activities of three citral-type (B1, B2, B3) and one non-citral type (B0) *C. bodinieri* varieties were 163.44 ± 0.84, 169.44 ± 0.09, 174.28 ± 2.31 and 104.26 ± 2.84, respectively. The alcohol dehydrogenase (*CbADH*) enzyme activities of B1, B2, B3 and B0 were 169.21 ± 1.76, 210.21 ± 2.02, 188.09 ± 0.50 and 136.38 ± 2.44, respectively. The enzyme activity of the geraniol synthase (*CbGES*) and alcohol dehydrogenase (*CbADH*) in the citral-type were higher than in non-citral type significantly by ELISA (*p* < 0.01) (Fig. 10).

## Discussion

The *C. bodinieri* is suitable for large-scale cultivation and convenient for harvesting in short rotation period, and rich in EOs, especially contain more than 80% citral in the citral-type, therefore, this plant has the potential to be an unrivalled source of natural citral [7]. The transcriptome is an effective tool for the study of terpene metabolite synthesis mechanisms, and the publication of *C. camphora* genome has provided good conditions for the study of the molecular mechanisms of Lauraceae plants [25, 27–31], and the reference transcriptome of *C. bodinieri* were first systemically investigated. The possible genes of citral formation was studied base on reference transcriptomics and metabolomics.

### Essential oils yield and composition of citral-type *C. Bodinieri*

While the leaves oil yield of citral-type *C. bodinieri* were 0.80–0.89%, the non-citral type were 0.35 ± 0.18%, indicating different chemotypes had significant differences (*p* ≤ 0.01) and the same chemotype with no significant difference. Similar field test was done in *C. bodinieri*, presented a different result that the non-citral type (1.18%) was higher than citral-type (0.26%) significantly [11]. In terms of the extract and the main components, there may be a significant difference among individuals, which were related to factors such as environmental, genetic, geographical, seasonal and extraction methods [10, 32, 33]. Therefore, we collected leaves from camphor trees grown in the same environment at the same time to ensure that the differences in results were only due to genetic factors. Metabolites were the basis of an organism’s phenotype and can contribute to understand biological processes and their mechanisms intuitively. We identified an interesting finding, monoterpene volatiles play important roles in aroma generation of *C. bodinieri*, forty-three constituents were identified comprising more than 90% of the composition of oils. The different chemical types of *C. bodinieri* do not differ morphologically, but differ in their main components. High content neral (32.57–37.13%) and geranial (48.06–49.15%) were identified in the essential oil of three citral-type (B1, B2, B3), which were higher than the rest of citral-rich plants in the world such as *Litsea cubeba* [10], *Cymbopogon citratus* [34], *Ocimum gratissimum* [35], *Backhousia citriodra* [36]. Those rare citral-type *C. bodinieri* varieties were identified through the scenting method from approximately 40,000 seedlings from Guizhou province, China. The citral-types were also high EOs yielding varieties, contained 80.63-86.33% citral and suitable for large-scale cultivation, therefore they will be expected to become new sources of natural citral. The study of the citral and camphor chemotype cannot only consider citral and camphor, but also the metabolic relationship of substances on the synthetic pathway of citral and camphor(Figure.3b, d) [37, 38]. Neral, geranial and geraniol were higher in citral-type, whereas geranic acid, (+)-borneol and (+)-camphor were higher in non-citral type of *C. bodinieri* (Table 2). These results illustrated the formation mechanism of citral and camphor of *C. bodinieri* at the level of substance metabolism.

### Terpene biosynthesis genes related to the formation of citral

In our research, the monoterpenes in the citral-type (87.70–92.28%) were higher than the non-citral type (75.8%)(Table 2), therefore, the high expression values (log(FPKM + 1)) of key genes *CbAACT*, *CbDXS*, *CbDXR*, *CbMVD*, *CbIPK*, *CbIDI*, *CbIPPI*, *CbGPPS* and *CbFDPS* of terpenoid backbone biosynthesis in citral-type were reasonable. The higher expression of these genes provided more precursors for downstream biosynthesis of monoterpenes, which were consistent with previous transcriptome results of *C. camphora* [18, 20], *Curcuma wenyujin* [39], *Litsea cubeba* [40], *tree peony* [41]. In many plants, geraniol synthase (*GES*) could catalyze geranyl pyrophosphate to generate geraniol [37, 42]. Besides, the biosynthesis of geraniol in rose is *RhNUDX1* gene, a new member of the Nudix hydrolase family [43, 44]. Geraniol and nerol are synthesized by the action of geraniol dehydrogenase or ethanol dehydrogenase [37, 45]. In our study, Geraniol dehydrogenase (*GDH*) was not identiffed from the transcriptome database, possibly due to incomplete annotation of structural genes in the reference genome, however the putative alcohol dehydrogenase (*CbADH*) gene expression in citral type were higher than the non-citral type. The enzyme activities of *CbGES* and *CbADH* in the citral-type were significantly higher than in the non-citral type by ELISA. Our research group have cloned the cDNA of the geraniol synthase from *C. camphora* (*CcGES*) and performed functional characterization of the protein encoded by *CcGES*. The terpene genes, such as cytochrome P450s (*CbCYP76B6* and *Cb10HGO*), terpene synthase (*CbATS*) and geranylgeranyl pyrophosphate synthase (*CbGPPS*), were significantly selected in the citral-type group but not in the non-citral type group. The cytochrome P450s is a class of enzymes necessary for the functionalization of monoterpenes, among which *CYP76B6* catalyze the hydroxylation of geraniol at their C-10 position to generate 8-oxogeraniol [46]. The *10HGO* was a key gene of iridoid glycoside synthesis, oxidizing 10-oxogeraniol to 10-oxogeranial, which was a NAPDH-dependent cytochrome P450 monoterpene oxidase [47, 48]. In our study, enhanced *CbCYP76B6* and *Cb10HGO* activity due to increased substrate as a result of increased geraniol content. Borneol, the precursor of camphor, was higher in non-citral type than in citral-type. Then it generated camphor by the catalytic of borneol dehydrogenase (*BDH*). Borneol diphosphate synthase (*CbBDH*) gene expression in the non-citral type were significantly higher than in the citral-types, which was associated with the synthesis of camphor [31, 38, 49, 50].

Although further analyses are needed to verificated by functional characterization of the protein encoded and genetic transformation experiments, this study nevertheless provides the molecular evidence for the *CbGES* and *CbADH* strong correlation with citral formation, as well as the *CbBDH* related to camphor formation in *C. bodinieri*.

## Conclusion

In recent years, high throughput sequencing and metabolome have provided several breakthroughs in aromatic plants. This work analyzed transcriptome and metabolite profiling of three citral-types and one non-citral type *C. bodinieri*. Monoterpenes were identified as main components in the leaves essential oils, neral and geranial were the most abundant components in the citral-type, while (+)-borneol and (+)-camphor were the major component in the non-citral type. Fifteen candidate unigenes about terpenoid biosynthesis were identified and validated. Among these genes, the *CbGES* and *CbADH* were correlated with citral formation, and the *CbBDH* was related to camphor formation in *C. bodinieri*, however, further functional studies are needed. This first reference transcriptome sequence of *C. bodinieri* database can contribute to understand the biosynthesis of citral in Lauraceae plants.

### Electronic supplementary material

Below is the link to the electronic supplementary material.

Supplementary Material 1

Supplementary Material 2

Supplementary Material 3

Supplementary Material 4

Supplementary Material 5

Supplementary Material 6

Supplementary Material 7

## Data Availability

Data supporting the findings of this work were available within the paper and its supplementary information files. Raw reads yielded from Illumina sequencing have been uploaded to the NCBI Sequence Read Archive (https://submit.ncbi.nlm.nih.gov/subs/sra/) and accession numbers for the twelve samples are as below: B1-1 (SAMN34992896); B1-2 (SAMN34992897); B1-3 (SAMN34992898); B2-1 (SAMN34992899); B2-2 (SAMN34992900); B2-3 (SAMN34992901); B3-1 (SAMN34992902); B3-2 (SAMN34992903); B3-3 (SAMN34992904); B0-1 (SAMN34992905); B0-2 (SAMN34992906); B0-3 (SAMN34992907).

## Declarations

### Material licences

The citral-type *C. bodinieri* used in our study were validated as Jiangxi provincial improved tree varieties after specificity, consistency and stability tests. The improved tree varieties belonged to Nanchang Institute of Technology and the breeders were Dr Qingyan Ling and Beihong Zhang. Our collection of *C. bodinieri* was approved by Nanchang Institute of Technology.
