# Supplementary figures and images for: Identification of key genes controlling monoterpene biosynthesis of Citral-type Cinnamomum bodinieri Levl. Based on transcriptome and metabolite profiling
Source: BMC Genomics. 2024 May 31;25:540. doi: 10.1186/s12864-024-10419-7 (PMC11141066; doi:10.1186/s12864-024-10419-7)

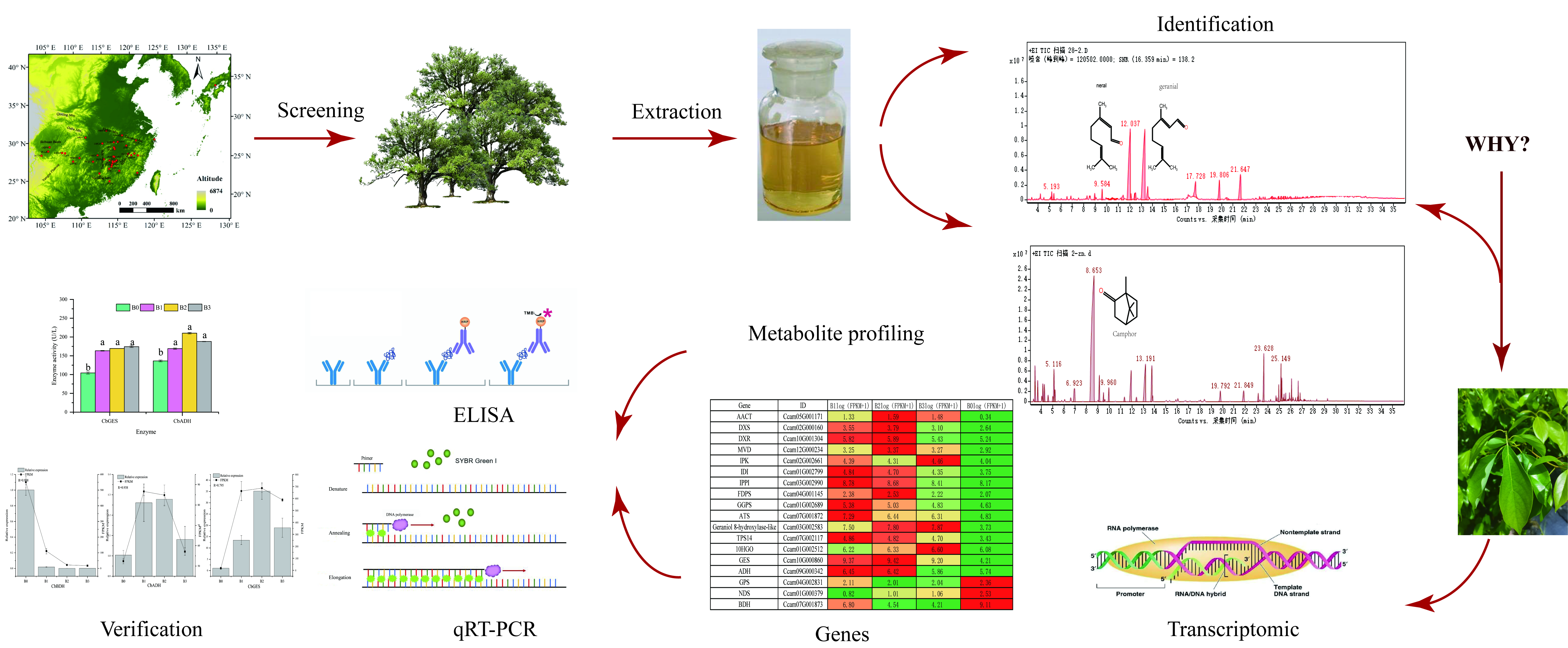

Supplement: Supplementary file 1 — Supplementary Material 1 [file 12864_2024_10419_MOESM1_ESM.jpg]
