# Supplementary material for: Identification of key genes controlling monoterpene biosynthesis of Citral-type Cinnamomum bodinieri Levl. Based on transcriptome and metabolite profiling
Source: BMC Genomics. 2024 May 31;25:540. doi: 10.1186/s12864-024-10419-7 (PMC11141066; doi:10.1186/s12864-024-10419-7)
Supplement: Supplementary file 2 — Supplementary Material 2 [file 12864_2024_10419_MOESM2_ESM.docx]

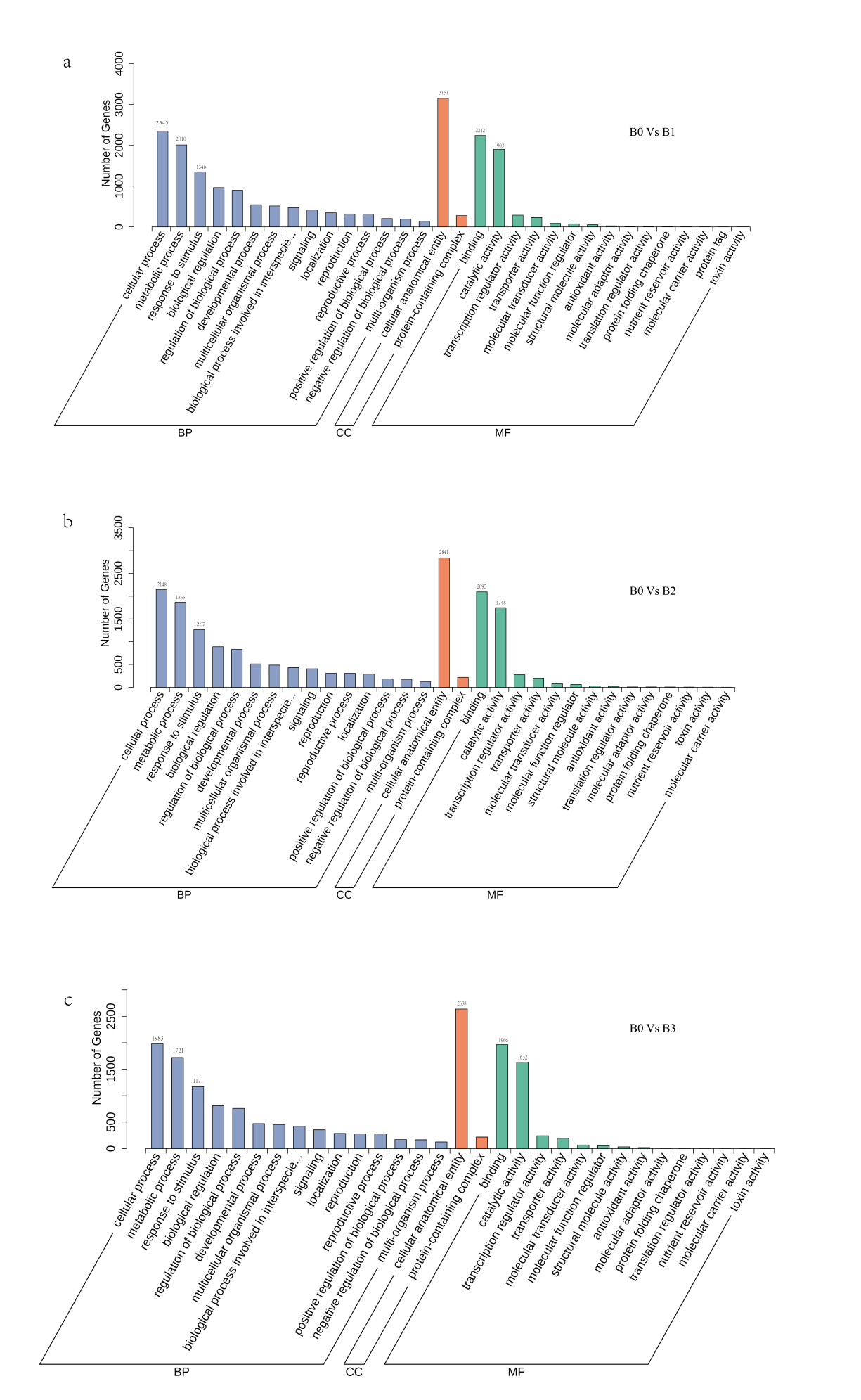
**Supplementary Figure S1.** Bar graph representing functional annotation based on gene ontology (GO) a B0 Vs B1 classification based on GO. b B0 Vs B2 classification based on GO. c B0 Vs B3 classification based on GO.


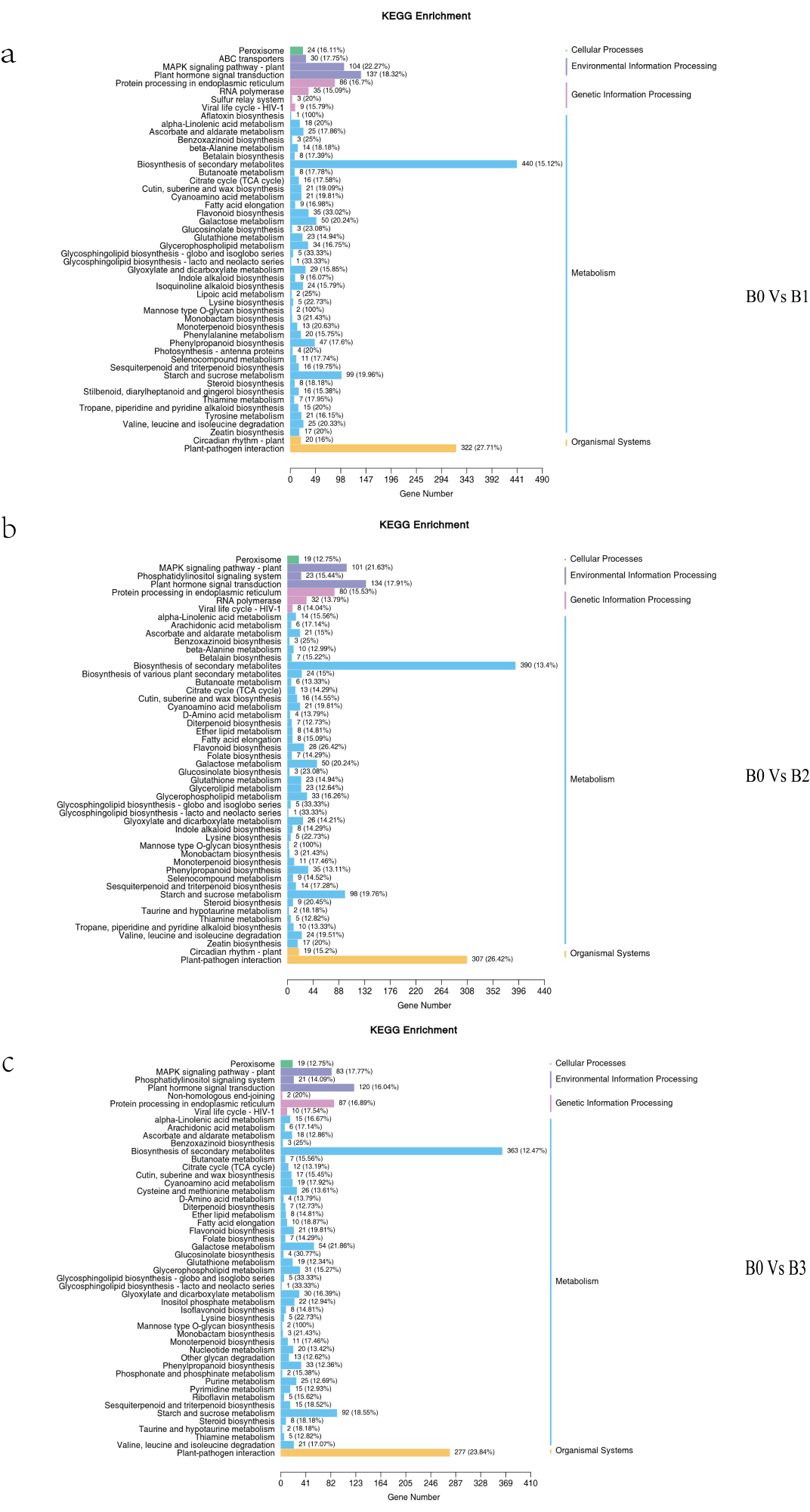


**Supplementary Figure S2.** Pathway enrichment bar based on Kyoto encylopaedia of genes and genomes (KEGG) a B0 Vs B1. b B0 Vs B2. c B0 Vs B3.
