# Supplementary material for: Identification of key genes controlling monoterpene biosynthesis of Citral-type Cinnamomum bodinieri Levl. Based on transcriptome and metabolite profiling
Source: BMC Genomics. 2024 May 31;25:540. doi: 10.1186/s12864-024-10419-7 (PMC11141066; doi:10.1186/s12864-024-10419-7)
Supplement: Supplementary file 3 — Supplementary Material 3 [file 12864_2024_10419_MOESM3_ESM.docx]

Supplementary Table S1 Summary of *C. bodinieri* transcriptome data

| Sample | Raw Reads | Clean Reads | Clean Base(G) | Q20(%) | Q30(%) | GC Content(%) | Mapped Reads |
| --- | --- | --- | --- | --- | --- | --- | --- |
| B1-1 | 50339458 | 49132022 | 7.37 | 97.34 | 92.42 | 46.42 | 41960419(85.40%) |
| B1-2 | 48374824 | 47196200 | 7.08 | 97.68 | 93.25 | 46.25 | 40365556(85.53%) |
| B1-3 | 46715594 | 44849688 | 6.73 | 97.54 | 92.91 | 46.1 | 37810200(84.30%) |
| B2-1 | 48737224 | 46877012 | 7.03 | 97.82 | 93.52 | 46.62 | 39739720(84.77%) |
| B2-2 | 48264314 | 46612974 | 6.99 | 97.47 | 92.77 | 46.89 | 39146356(83.98%) |
| B2-3 | 46049162 | 44275836 | 6.64 | 97.7 | 93.24 | 46.19 | 37217320(84.06%) |
| B3-1 | 43327532 | 41624756 | 6.24 | 97.78 | 93.45 | 46.31 | 35317905(84.85%) |
| B3-2 | 45825936 | 44211450 | 6.63 | 97.71 | 93.28 | 46.3 | 37491843(84.80%) |
| B3-3 | 45418678 | 43598510 | 6.54 | 97.57 | 92.93 | 46.69 | 37005390(84.88%) |
| B0-1 | 46382810 | 44238890 | 6.64 | 97.61 | 93.07 | 46.68 | 37116385(83.90%) |
| B0-2 | 43203692 | 41047378 | 6.16 | 97.42 | 92.63 | 46.51 | 34174002(83.26%) |
| B0-3 | 46197036 | 44357816 | 6.65 | 97.25 | 92.27 | 46.91 | 37200457(83.86%) |

Supplementary Table S2 Primers sequences used for qRT-PCR

| Primers name | Forward primer sequence（5’-3’） | Reverse primer sequence（5’-3’） |
| --- | --- | --- |
| CbAACT | TACTGCAAACAGCCCCGTTC | AGGAATCCTCCCATCGGTGT |
| CbDXS | GGACTCAAGCCTTTCTGTGC | TAGCAACCATGTGCACCAAT |
| CbDXR | TTGCAGATCAGGTGAAGACG | GGTGACGTGCAACTTCAATG |
| CbMVD | CGCGAGAGTGTTGACACAAG | GGGAGGAGAGGTATCCAAGC |
| CbIpK | GAGCAGGTTCTTTTGGGCAC | AGTGCCCTCACGATCTCAAG |
| CbGGPS | TCATGCTGGCTGCTTCCTAC | AGCTCGAACCCAAGAGGGTA |
| CbFDPS | ACCGGGTTCAGAGCATGTTG | TGCACTGATTTGCTTGGCAG |
| CbGES | ATGCCACTCCTTGTTGGAGG | CCTGAGTGGACCCTGTTTGT |
| CbADH | GTTCCTCACAAGGATGCCGT | ACAGAAGGCAGATCAGAGCG |
| CbCYP76B6-like | ACATACGGCCCACTGATGAC | ATACCCACCAATCGCAAGCA |
| Cb10HGO | ACGAATGGGGAATGTCCGTC | CAACACCCGCCTTATCTCCA |
| CbATS-1 | TTGAACTGCCTTCCAAGCTCT | AACTTCTCAGCTCGTTTGGC |
| CbATS-2 | GCTAAGGCTGGAGGCTAGGT | CTCCACCAGCCTATCTCTCG |
| CbNDS | AATCTTGGGGCTGATCCGTTC | GACGCCCCATTTCAGAGGA |
| CbBDH | CACATCGTTGCTGGTTCGAC | AACACCGTCCAATCCGAGAG |

Supplementary Table S3 Differential expression genes (DEGs) annotation results

| Databases | DEGs number |
| --- | --- |
| NR | 5560 |
| Pfam | 4697 |
| Swiss-prot | 4357 |
| TF | 418 |
| KEGG | 4285 |
| GO | 4842 |
| Trembl | 5555 |
| KOG | 2868 |
| Total | 5966 |
